# Genetic Variations in the Human Cannabinoid Receptor Gene Are Associated with Happiness

**DOI:** 10.1371/journal.pone.0093771

**Published:** 2014-04-01

**Authors:** Masahiro Matsunaga, Tokiko Isowa, Kaori Yamakawa, Seisuke Fukuyama, Jun Shinoda, Jitsuhiro Yamada, Hideki Ohira

**Affiliations:** 1 Department of Health and Psychosocial Medicine, Aichi Medical University School of Medicine, Aichi, Japan; 2 Department of Gerontological Nursing, Mie University Faculty of Medicine, Mie, Japan; 3 Department of Psychology, Graduate School of Environmental Studies, Nagoya University, Aichi, Japan; 4 Chubu Medical Center for Prolonged Traumatic Brain Dysfunction, Kizawa Memorial Hospital, Gifu, Japan; Hospital General Dr. Manuel Gea González, Mexico

## Abstract

Happiness has been viewed as a temporary emotional state (e.g., pleasure) and a relatively stable state of being happy (subjective happiness level). As previous studies demonstrated that individuals with high subjective happiness level rated their current affective states more positively when they experience positive events, these two aspects of happiness are interrelated. According to a recent neuroimaging study, the cytosine to thymine single-nucleotide polymorphism of the human cannabinoid receptor 1 gene is associated with sensitivity to positive emotional stimuli. Thus, we hypothesized that our genetic traits, such as the human cannabinoid receptor 1 genotypes, are closely related to the two aspects of happiness. In Experiment 1, 198 healthy volunteers were used to compare the subjective happiness level between cytosine allele carriers and thymine-thymine carriers of the human cannabinoid receptor 1 gene. In Experiment 2, we used positron emission tomography with 20 healthy participants to compare the brain responses to positive emotional stimuli of cytosine allele carriers to that of thymine-thymine carriers. Compared to thymine-thymine carriers, cytosine allele carriers have a higher subjective happiness level. Regression analysis indicated that the cytosine allele is significantly associated with subjective happiness level. The positive mood after watching a positive film was significantly higher for the cytosine allele carriers compared to the thymine-thymine carriers. Positive emotion-related brain region such as the medial prefrontal cortex was significantly activated when the cytosine allele carriers watched the positive film compared to the thymine-thymine carriers. Thus, the human cannabinoid receptor 1 genotypes are closely related to two aspects of happiness. Compared to thymine-thymine carriers, the cytosine allele carriers of the human cannabinoid receptor 1 gene, who are sensitive to positive emotional stimuli, exhibited greater magnitude positive emotions when they experienced positive events and had a higher subjective happiness level.

## Introduction

Happiness is one of the most fundamental human goals, which has led researchers to examine the source of individual happiness. Happiness as a scientific construct has been viewed as a temporary emotional state (hedonia) and a relatively stable state of being happy (eudaimonia) [1]–[3]. Hedonia is usually experienced when we get the material objects and action opportunities we wish to possess or experience [4]–[8]. Therefore, at the least, hedonia corresponds to a psychological state of pleasure and is greatly influenced by life events or circumstances, such as health, human relationships, household income, and housing conditions [3]–[6]. In contrast, according to previous studies, there is a relatively stable state of being happy, which is related to the sum of one's recent levels of affect, one's satisfaction with life, and disposition/propensity [1]–[3]. Although eudaimonia may be more difficult to define scientifically, at the least, it corresponds to a subjective assessment of one's life lived well (e.g., subjective well-being or subjective happiness level) rather than to an emotional feeling [4]–[6], [9].

Although there is a conceptual distinction between pleasure and subjective happiness level, these two aspects of happiness are interrelated. According to previous studies, individuals with a high subjective happiness level rated their current affective states more positively when they experience positive events [4], [10], [11]. Conversely, consecutive experiences of positive events also increased their subjective happiness level [4], [7], [8]. Psychologists have established two psychological models that explain the relationship between these two aspects of happiness: a top-down and a bottom-up model [3], [4]. The top-down model assumes that individuals with a positive propensity, such as optimism, may evaluate their subjective happiness level and daily life events more positively than others who experience a similar number of positive life events [3], [4]. In contrast, the bottom-up model suggests that hedonic experience in each of the life domains (e.g., household income, housing conditions) influence subjective happiness level positively [3], [4]. Thus, the sum of positive life events may be important for constructing a subjective happiness level. However, previous epidemiological data have suggested a more complex interaction between these two happiness components; there is both top-down and bottom-up processes in happiness processing [3], [4]. Thus, researchers have proposed a third psychological model, the up-down model, which allows for bidirectional interactions between the two happiness components [3], [4].

Recent studies have suggested that the human endocannabinoid system is associated with positive emotional processing [12]. The endocannabinoid system refers to a group of neuromodulatory lipids [13]. The endocannabinoids such as anandamide and 2-arachidonoyl glycerol [14]–[16] bind to brain cannabinoid receptors, which are involved in several physiological processes including appetite, nociception, mood, and memory [13]. The function of cannabinoid receptor 1 (CB1) is well known. In the brain, CB1 inhibits γ-aminobutyric acid (GABA) transmission presynaptically [17], [18]. Recent studies have demonstrated a role for CB1 in modulating the brain reward system; in fact, CB1 activation may induce dopamine release in the striatum including the nucleus accumbens [19], [20]. The human CB1 receptor gene (*CNR1*) is located at chromosome 6q14–15 and there are multiple single-nucleotide polymorphisms (SNPs) in *CNR1*; however, its effects on CB1 receptor function are still unclear [21]. A recent study indicated that a cytosine to thymine (C/T) SNP of *CNR1* (dbSNP number rs 806377), located in the untranslated exon 3, modulated striatal responses to happy faces [12]. The striatal activity in the cytosine (C) allele carriers was higher than that in individuals with a thymine-thymine (TT) genotype when happy faces were presented [12], suggesting that the C allele of the SNP of the *CNR1* may enhance CB1 activity.

Based on these previous observations, our genetic traits, such as the *CNR1* genotypes, are closely related to the two aspects of happiness. That is, compared to individuals with TT genotypes, C allele carriers of the SNP of *CNR1*, who are sensitive to positive emotional stimuli, may experience a higher magnitude pleasure response when they experience positive events and may have a higher subjective happiness level. In Experiment 1, to investigate the difference in subjective happiness level between C allele carriers and TT carriers, we assessed the subjective happiness level of 198 healthy participants. In Experiment 1, the participants were asked to evaluate their subjective happiness level using the Japanese version of the Subjective Happiness Scale (JSHS) [10], [11], [22], which included a subjective assessment of general happiness level and a subjective assessment of one's positive personal trait [9]. We predicted that subjective happiness level was higher in C allele carriers compared to TT carriers. Furthermore, in Experiment 2, we investigated the differences in brain responses and subjective ratings of temporal mood states between C allele carriers and TT carriers. To do so, we conducted a positron emission tomography (PET) study with an independent group of 20 healthy participants using the task of looking at one's favorite persons. Our previous PET study using the same task indicated that the act of looking at one's favorite persons evoked positive emotions [10]. Activation of the medial prefrontal cortex (mPFC) was positively correlated with the participants' self-rating of current positive mood states [10]. Thus, compared to TT carriers, we predicted that C allele carriers may evaluate their current mood state more positively and their mPFC may be activated to a greater degree when they looked at their favorite persons. Fortunately, the aim of this study was achieved through these experiments.

## Materials and Methods

### Experiment 1

#### Participants

We recruited 198 healthy volunteers (76 males and 122 females; age range: 18–40 years; age mean, 23.2±0.37 (standard error of the mean: SEM) years), following study approval by the Ethics Committee of Fujita Health University (previous affiliation of the first author; approval number: 09-018). All participants provided written informed consent in accordance with the Declaration of Helsinki. Participants were excluded if they had a chronic and infectious illness or if they had taken medication during the week prior to the experiment. All participants were Japanese undergraduate and graduate students of universities near our university.

#### Assessment of subjective happiness level

We used the JSHS to assess subjective happiness level of the participants [10], [11], [22]. The JSHS is a 4-item scale that measures relatively stable subjective happiness. Each item is evaluated by a 7-point Likert scale and the middle point (4) represents neither unhappy nor happy. The internal consistency, test-retest reliability, convergent validity, and discriminant validity of the JSHS have previously been confirmed [9]–[11], [22]. The Cronbach's α value of the JSHS was 0.82 in the original paper [22]. The mean JSHS score for the participants was 4.84±0.07 (SEM), which was similar to that reported previously [10], [11], [22], indicating that distribution of subjective happiness level in the participants was similar to that reported previously [10], [11], [22].

#### Genotyping

Genomic DNA was extracted from peripheral blood collected from the participants by using a DNA Extractor WB-Rapid Kit (Wako, Osaka, Japan). The SNP marker for the *CNR1* (dbSNP number rs806377) was genotyped using TaqMan® SNP Genotyping Assays (Applied Biosystems, Foster City, CA), which were functionally tested by Applied Biosystems and available on demand. All polymerase chain reactions (PCR) and allelic discrimination reactions were performed on a StepOne PlusTM Real-Time PCR System (Applied Biosystems). Consistent with a previous study [23], the proportion of those with the cytosine-cytosine (CC) genotype was very small in this population (21 CC (10.6%), 86 cytosine-thymine (CT) (43.4%), and 91 TT (46.0%)). Thus, we grouped the CC and CT carriers together (C allele carriers: *n* = 107)) and compared their subjective happiness level to that of the TT carriers (*n* = 91).

#### Statistical analyses

The group subjective happiness levels were expressed as the means ± standard error of the mean (SEM). We used a Student's *t*-test to compare the subjective happiness level of the C allele carriers and the TT carriers. Further, the following regression model was employed to test for genetic associations:
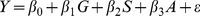



In this case, “*G*” is a matrix of variables to control for the *CNR1* genotype, whereby *G* = 1 if the participant's genotype was CC or CT, and *G* = 0 if the genotype was TT. “*S*” is a matrix of variables to control for sex (*S* = 1 if the participant was male and *S* = 2 if the participant was female). “*A*” is a matrix of variables to control for age, and “*ε*” is an individual-specific error.

### Experiment 2

#### Participants

We recruited 20 healthy male right-handed volunteers (age range: 20–30 years; age mean, 22.2±0.65 (SEM) years), following study approval by the Ethics Committee of Aichi Medical University (approval number: 413). Only men were studied to avoid potential influences of the menstrual cycle on the physiological responses of women. All participants provided written informed consent in accordance with the Declaration of Helsinki. Participants were excluded if they had a chronic and infectious illness or if they had taken medication during the week prior to the experiment. All participants were Japanese undergraduate and graduate students of universities near our university. The mean body mass index (BMI) of the participants was 21.5±0.71 (SEM), indicating that the participants had a normal weight. We divided the participants into the TT carriers (*n* = 10) and the CT and CC carriers (C allele carriers: *n* = 10) by using the genotyping technique that was similar to that used in Experiment 1. No between-group differences in age and BMI were detected.

#### Experimental Procedure

The experimental procedure used in the present PET study was similar to that used in previous studies [10], [24]. In brief, participants were instructed not to eat 2 h before the scanning session, but they were allowed to consume non-alcoholic and caffeine-free fluids. Either an emotionally neutral film (control film) or a film featuring people the participants considered attractive (positive film) was presented for 4 min on a 15-inch display positioned approximately 60 cm away from the participants. The PET (duration: 60 s) scans were performed in the first 2–3 min of the screening. Mood states were evaluated before and after the screening. To obtain reliable PET data, each participant was assigned two distinct positive and two distinct control films, and four PET scans were obtained per participant. In order to assess the mood states of participants, they were asked to rate how positive their mood was before and after watching the films on the Visual Analog Scale (VAS). The VAS ranged from 0% (not positive) to 100% (extremely positive).

#### Experimental stimuli

We compiled 4-min audiovisual clips. The positive film featured a person whom each participant subjectively considered attractive. By free response, the participants themselves selected this person before the day of the experiment. All the selected persons were famous actresses. By the day of the experiment, we compiled an individual 4-min video film from television (TV) programs and movies for each participant. In order to obtain the maximum effect, we did not standardize the actions performed by the actresses in the movies; the films did not contain erotic or sexually suggestive scenes. For example, one film contained scenes of their favorite person smiling. In addition, we compiled audiovisual clips because we thought that the favorite person's voice was important for the participants. The control film was a TV news program with a newscaster whom participants considered not so attractive. Since the newscaster reported past weather events, rather than new information, the participants were uninterested in the film. The participants watched the edited control and positive films for the first time at the time of the experiment. The emotional valence of the films was evaluated previously [10], [24].

#### Image Acquisition by Positron Emission Tomography

During each block, the distribution of regional cerebral blood flow (rCBF) was measured by a General Electric Advance NXi PET scanner (GE Healthcare Life Sciences, Little Chalfont, England) operated in a high-sensitivity three-dimensional mode. A venous catheter (for administering the tracer) was inserted into the antecubital fossa vein of the left forearm. After the subject's head was positioned in an inflatable plastic head holder to prevent possible head movements, a 10-min transmission scan using a rotating ^68^Ge pin source was completed. In each block, after a 370-MBq bolus injection of H_2_^15^O over 30 s, scanning was started and continued for 60 s. Bolus injection was started 60 s after the initiation of the block. The integrated radioactivity accumulated during the 60 s of scanning was used as an index of rCBF. Six scans were acquired per subject. The interval between successive scans was 15 min, to allow radioactivity levels to return to baseline. A Hanning filter was used to reconstruct images into 35 planes with a thickness of 4.5 mm and a resolution of 2×2 mm (full width at half maximum).

#### Image Processing and Analysis

We used SPM8 revision 5236 (The Wellcome Trust Centre for Neuroimaging; http://www.fil.ion.ucl.ac.uk/spm) in MATLAB 2013a (MathWorks Inc., Natick, MA, USA) to analyze functional images. The images were initially realigned using sinc interpolation to remove artifacts before they were transformed into a standard stereotactic space. Images were corrected for whole-brain global blood flow by proportional scaling and were smoothed using a Gaussian kernel to a final in-plane resolution of 8 mm at full width at half maximum. In order to delete the activations in the non-emotional brain regions, such as the visual and auditory cortices, the PET images obtained during the control condition were subtracted from those obtained during the positive condition. Differences within genotypes (CC + CT versus TT) were the primary focus of the present study. Thus, the images in four conditions (CC + CT (positive − control) − TT (positive − control)) were incorporated into a general linear model [25]. Voxel values for each contrast yielded a statistical parametric map of the *t* statistic (Statistical Parameter Mapping (SPM) *t*), which was subsequently transformed to a unit normal distribution (SPM *z*). Based on our prior PET results [10], the mPFC was hypothesized a priori to be involved in positive emotion evocation in the present psychological task. Thus, the region of interest (ROI) analysis was performed. Anatomical mPFC ROI was defined using the Automated Anatomical Labeling (AAL) atlas [26] from the Wake Forest University (WFU) Pickatlas [27] integrated in SPM8. To characterize activation in the ROI (mPFC), the statistical threshold was set at an uncorrected p<0.001 at the voxel level and a family-wise error (FWE) corrected p<0.05 at the cluster level.

## Results

### Difference in subjective happiness level (Experiment 1)

**Figure 1:**
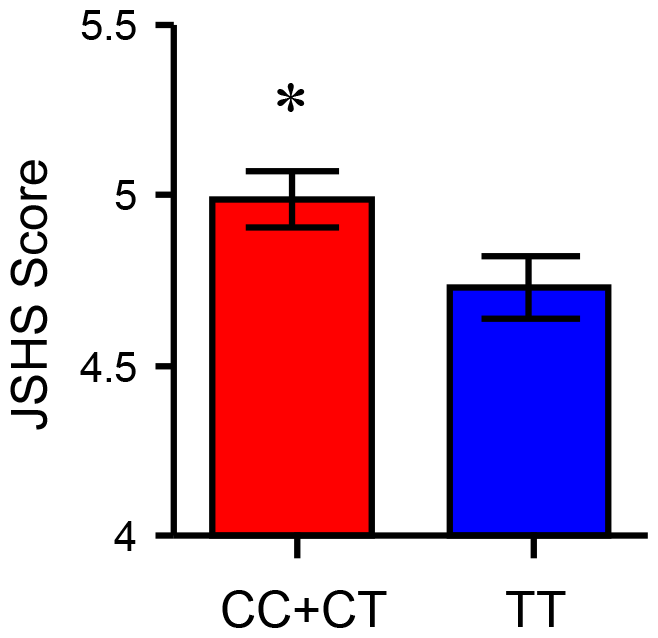
Subjective happiness level comparisons between *CNR1* CC + CT (C allele carriers) and TT carriers. Each column and vertical line represents the mean ± standard error of the mean. *p<0.05 versus TT carriers, Student's *t*-test. JSHS: Japanese version of subjective happiness scale.

Figure 1 illustrates the subjective happiness level difference between the C allele carriers and the TT carriers. The statistical analysis revealed that the JSHS score of the C allele carriers was significantly higher than that of the TT carriers (df = 196, *t* = 2.07, *p*<0.05; two-tailed). Table 1 depicts the results of the regression model, which tested the hypothesis that variations in *CNR1* are associated with subjective happiness. The model included age and gender variables. It revealed that the C allele of *CNR1* was significantly associated with an increased subjective happiness level (*p*<0.05).

**Table 1:** Results from the regression analysis examining the association between *CNR1* genotypes.

Predictor variables	*β*	*t*	*p*-value
*CNR1* C allele	0.140	1.982	<0.05
Sex	0.086	1.203	0.231
Age	−0.035	−0.497	0.620
*N*	198		
*Adjusted R^2^*	0.02		

All predictor variables were included in the regression analysis. *β*, Standardized beta coefficients.

### Differences in psychological and brain responses to positive stimuli (Experiment 2)

**Figure 2:**
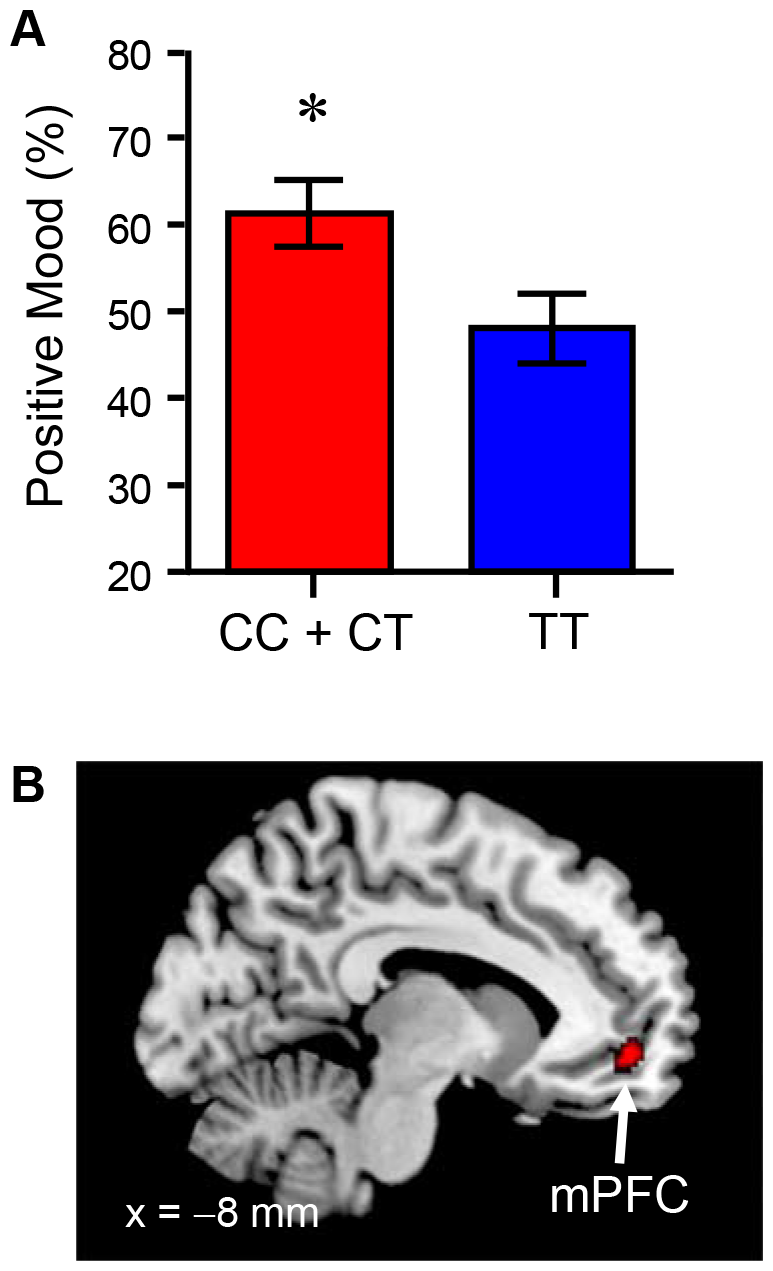
Comparisons of psychological and brain responses to positive stimuli between *CNR1* genotypes. (A) The difference in positive mood rating scores after watching the positive film between the CC + CT (C allele carriers) and the TT carriers. Each column and vertical line represents the mean ± standard error of the mean. *p<0.05 versus TT carriers, Student's *t*-test. (B) A statistical parametric map showing a significant cluster that, compared to the TT carriers, was activated more strongly in C allele carriers when they watched the positive film. The statistical threshold for the analysis was set at *p*<0.001 (uncorrected) at the voxel level and *p*<0.05 (corrected) at the cluster level. mPFC: medial prefrontal cortex.

Figure 2a illustrates the positive mood differences between the C allele carriers and the TT carriers after they watched the positive film. The statistical analysis revealed that the positive mood rating score after watching the positive film was significantly higher for the C allele carriers than the TT carriers (df = 18, *t* = 2.33, *p*<0.05; two-tailed). Prior to watching the film, there was no between-groups difference in positive mood (df = 18, *t* = 1.25, *p* = 0.22; two-tailed). Furthermore, we conducted a subtraction analysis to reveal the brain regions that were strongly activated in the positive condition in the C allele carriers. The subtraction analysis indicated that, compared to the TT carriers, the mPFC (peak coordinates: x = −8, y = 50, z = −8; p<0.05 corrected for multiple comparisons within the ROI; Cluster size = 28; *t* = 3.71) was activated to a greater degree in the C allele carriers (Figure 2b).

## Discussion

In this study, we hypothesized that the *CNR1* genotypes were closely related to happiness. That is, compared to the TT carriers, the C allele carriers of the SNP of the *CNR1* (rs806377), who are sensitive to positive emotional stimuli, may experience greater magnitude positive emotions when they experience positive events and may have a higher subjective happiness level.

### Association between *CNR1* genotypes and subjective happiness level

Experiment 1 indicated that subjective happiness level in C allele carriers was significantly higher than that in TT carriers (Figure 1). The regression analysis, which included age and gender variables, indicated that the C allele of *CNR1* is significantly associated with subjective happiness levels in Japanese adults (Table 1). Although it should be noted that, compared to the current standards of behavioral genetics, this was a small-scale experiment, our results indicated that the C allele carriers of *CNR1* have a higher subjective happiness level compared to the TT carriers. This is consistent with our a priori hypothesis.

### Associations between *CNR1* genotypes and responses to positive stimuli

The results of Experiment 2 indicated that the positive mood after watching the positive film was significantly higher in the C allele carriers than the TT carriers (Figure 2a). Subtraction analysis of PET images indicated that, compared to the TT carriers, the C allele carriers exhibited greater activation of the mPFC when they watched the positive film (Figure 2b). Recent neuroimaging studies indicated that the mPFC is involved in the evaluation of self-related emotional events [28]–[31]. The dorsal part of the mPFC is involved in the monitoring of one' behaviors that enable the selection of goal-directed event sequences; the ventral part of the mPFC is involved in reward (outcome) processing [28]–[31]. The most rostral part of the mPFC allows the integration of information from the dorsal goal pathway with information from the ventral outcome pathway [30]. Thus, activation of the rostral mPFC may represent personal goal attainment. The emotional stimuli of the present PET study were the participant's personal favorite actresses (self-referential positive stimuli), suggesting that looking at their favorite persons may be linked to personal goal attainment. Thus, activation of the mPFC by looking at their favorite persons represents positive emotion evocation. In fact, our previous PET study indicated that the degree of mPFC activation is positively correlated with positive mood rating scores [10]. Therefore, the present PET study indicated that, compared to the TT carriers, positive emotion-related brain region was activated to a greater extent in the C allele carriers when they watched the positive film, although there were fewer participants in the present study (i.e., 20 participants) compared to the current standards of neuroimaging studies. Considering all the information, compared to the TT carriers, the C allele carriers exhibited a higher sensitivity to positive emotional stimuli. This is consistent with our a priori hypothesis.

### Hedonic brain circuit and the endocannabinoid system

Berridge and Kringelbach [6] previously discussed the interaction between the endocannabinoid system and pleasure generators in the brain (hedonic hotspots), such as the nucleus accumbens and ventral pallidum. In rodent studies, the nucleus accumbens can be activated by microinjection of the endocannabinoid (anandamide); anandamide microinjections into the nucleus accumbens doubled the number of positive “liking” reactions elicited by intraoral sucrose [32]. In humans, a previous study indicated that the activity of the globus pallidus in C allele carriers of *CNR1* was higher than that in TT carriers when happy faces were presented [12]. Thus, the endocannabinoid system may positively modulate the sensitivity to positive emotional stimuli through the modulation of hedonic brain hotspot functions. Although we did not focus on the activation of the striatum in the present PET study, several neuroimaging studies have shown that activity in the mPFC correlates with the activity in the striatum [33], [34]. Thus, it is possible that higher activation of the striatum or strong connectivity between the striatum and the mPFC in the C allele carriers is observed by using the present psychological task. To test this hypothesis, we will have to conduct a further neuroimaging study in future.

### Happiness and the endocannabinoid system

According to the psychological bottom-up model of subjective well-being, hedonic experiences in each life domain had a positive influence on subjective happiness level [3], [4]. Thus, the sum of positive life events may be important for constructing subjective happiness level. Because C allele carriers of *CNR1* have a high sensitivity to positive emotional events, positive life events may be more abundant in C allele carriers than TT carriers. Therefore, it is possible that the *CNR1* genotype is associated with subjective happiness level by means of abundant positive life events. In contrast, the top-down model of subjective well-being assumes that individuals with a positive propensity may evaluate their subjective happiness level and daily life events more positively than others who experience a similar number of positive life events [3], [4]. To the best of our knowledge, the association between trait optimism and *CNR1* has not yet been examined; however, it is possible that our genetic traits are associated with our personality traits. In fact, a previous neuroimaging study demonstrated that the degree of trait optimism was positively correlated with the response of the rostral anterior cingulate cortex (rACC) to positive stimuli [35]. Although the rACC is anatomically divided from the mPFC by the cingulate sulcus, these prefrontal cortices are interrelated through the cingulate fasciculus [36]–[38]. The rACC is an affective division of the anterior cingulate cortex (ACC) [36], [37]. Previous studies showed that the rACC is engaged during emotional conflict resolution and associated with positive emotional states through the inhibition of maladaptive activation of the amygdala [36], [37]. Our present study indicated that the rostral mPFC of C allele carriers was strongly activated by positive emotional stimuli, suggesting that *CNR1* might be associated with trait optimism. Therefore, it is possible that the *CNR1* genotype is associated with subjective happiness level by means of trait optimism.

### Conclusions

The present study indicated that our genetic traits, the *CNR1* genotypes, are closely related to happiness. Compared to the TT carriers, the C allele carriers of the SNP of the *CNR1* (rs806377), who are sensitive to positive emotional stimuli, exhibited greater magnitude positive emotions when they experience positive events and have a higher subjective happiness level. The present findings can applied to several scientific fields such as psychology, biology, clinical, and pedagogical fields.

## References

[1] Kozma A, Stones MJ (1983) Predictors of happiness. J Gerontology 38: 626–628. 10.1093/geronj/38.5.626 6886321

[2] Kozma A, Stone S, Stones MJ, Hannah TE, McNeil K (1990) Long- and short-term affective states in happiness: model, paradigm and experimental evidence. Soc Indic Res 22: 119–138.

[3] Stones MJ, Hadjistavropoulos T, Tuuko H, Kozma A (1995) Happiness has traitlike and statelike properties: A reply to Veenhoven. Soc Indic Res 36: 129–144.

[4] Schimmack U (2008) The structure of subjective wellbeing. In: Eid M, Larsen RJ, editors. The science of subjective well-being.New York: Guilford Press, pp. 97–123.

[5] Waterman AS, Schwartz SJ, Conti R (2008) The implications of two conceptions of happiness (hedonic enjoyment and eudaimonia) for the understanding of intrinsic motivation. J Happiness Stud 9: 41–79.

[6] Berridge KC, Kringelbach ML (2011) Building a neuroscience of pleasure and well-being. Psychol Well Being 1: 1–3. 22328976 10.1186/2211-1522-1-3 PMC3274778

[7] Seligman ME, Steen TA, Park N, Peterson C (2005) Positive psychology progress: empirical validation of interventions. Am Psychol 60: 410–421. 16045394 10.1037/0003-066X.60.5.410

[8] Otake K, Shimai S, Tanaka-Matsumi J, Otsui K, Fredrickson BL (2006) Happy people become happier through kindness: a counting kindnesses intervention. J Happiness Stud 7: 361–375. 17356687 10.1007/s10902-005-3650-z PMC1820947

[9] Lyubomirsky S, Lepper HS (1999) A measure of subjective happiness: Preliminary reliability and construct validation. Soc Indic Res 46: 137–155.

[10] Matsunaga M, Murakami H, Yamakawa K, Isowa T, Fukuyama S, et al (2011) Perceived happiness level influences evocation of positive emotions. Natural Science 3: 723–727 10.4236/ns.2011.38095

[11] Matsunaga M, Isowa T, Yamakawa K, Tsuboi H, Kawanishi Y, et al (2011) Association between perceived happiness levels and peripheral circulating pro-inflammatory cytokine levels in middle-aged adults in Japan. Neuro Endocrinol Lett 32: 458–463. 21876513

[12] Chakrabarti B, Kent L, Suckling J, Bullmore E, Baron-Cohen S (2006) Variations in the human cannabinoid receptor (CNR1) gene modulate striatal responses to happy faces. Eur J Neurosci 23: 1944–1948. 16623851 10.1111/j.1460-9568.2006.04697.x

[13] Moreira FA, Lutz B (2008) The endocannabinoid system: emotion, learning and addiction. Addict Biol 13: , 196–212. doi: 10.1111/j.1369-1600.2008.00104.x. 10.1111/j.1369-1600.2008.00104.x 18422832

[14] Devane WA, Axelrod J (1994) Enzymatic synthesis of anandamide, an endogenous ligand for the cannabinoid receptor, by brain membranes. Proc Natl Acad Sci USA 91: 6698–6701. 8022836 10.1073/pnas.91.14.6698 PMC44270

[15] Stella N, Schweitzer P, Piomelli D (1997) A second endogenous cannabinoid that modulates long-term potentiation. Nature 388: 773–778. 9285589 10.1038/42015

[16] Okamoto Y, Wang J, Morishita J, Ueda N (2007) Biosynthetic pathways of the endocannabinoid anandamide. Chem Biodivers 4: 1842–1857. 17712822 10.1002/cbdv.200790155

[17] Katona I, Rancz EA, Acsady L, Ledent C, Mackie K, et al (2001) Distribution of CB1 cannabinoid receptors in the amygdala and their role in the control of GABAergic transmission. J Neurosci 21: 9506–9518. 11717385 10.1523/JNEUROSCI.21-23-09506.2001 PMC6763903

[18] Hoffman AF, Riegel AC, Lupica CR (2003) Functional localization of cannabinoid receptors and endogenous cannabinoid production in distinct neuron populations of the hippocampus. Eur J Neurosci 18: 524–534. 12911748 10.1046/j.1460-9568.2003.02773.x

[19] van der Stelt M, Di Marzo V (2003) The endocannabinoid system in the basal ganglia and in the mesolimbic reward system: implications for neurological and psychiatric disorders. Eur J Pharmacol 480: 133–150. 14623357 10.1016/j.ejphar.2003.08.101

[20] Sperlágh B, Windisch K, Andó RD, Sylvester Vizi E (2009) Neurochemical evidence that stimulation of CB1 cannabinoid receptors on GABAergic nerve terminals activates the dopaminergic reward system by increasing dopamine release in the rat nucleus accumbens. Neurochem Int 54: 452–457 10.1016/j.neuint.2009.01.017 19428788

[21] Zhang PW, Ishiguro H, Ohtsuki T, Hess J, Carillo F, et al (2004) Human cannabinoid receptor 1: 5′ exons, candidate regulatory regions, polymorphisms, haplotypes and association with polysubstance abuse. Mol Psychiatry 9: 916–931. 15289816 10.1038/sj.mp.4001560

[22] Shimai S, Otake K, Utsuki N, Ikemi A, Lyubomirsky S (2004) Development of a Japanese version of the subjective happiness scale (SHS), and examination of its validity and reliability. Japanese Journal of Public Health 51: 845–853. 15565993

[23] Mitjans M, Serretti A, Fabbri C, Gastó C, Catalán R, et al (2013) Screening genetic variability at the CNR1 gene in both major depression etiology and clinical response to citalopram treatment. Psychopharmacology (Berl) 227: 509–519 10.1007/s00213-013-2995-y 23407780

[24] Matsunaga M, Isowa T, Kimura K, Miyakoshi M, Kanayama N, et al (2008) Associations among central nervous, endocrine, and immune activities when positive emotions are elicited by looking at a favourite person. Brain Behav Immun 22: 408–417. 17977695 10.1016/j.bbi.2007.09.008

[25] Friston K J (2007) Statistical parametric mapping. London: Academic Press. 679 p.

[26] Tzourio-Mazoyer N, Landeau B, Papathanassiou D, Crivello F, Etard O, et al (2002) Automated anatomical labeling of activations in SPM using a macroscopic anatomical parcellation of the MNI MRI single-subject brain. Neuroimage 15: 273–289. 11771995 10.1006/nimg.2001.0978

[27] Maldjian JA, Laurienti PJ, Kraft RA, Burdette JH (2003) An automated method for neuroanatomic and cytoarchitectonic atlas-based interrogation of fMRI data sets. Neuroimage 19: 1233–1239. 12880848 10.1016/s1053-8119(03)00169-1

[28] Roy M, Shohamy D, Wager TD (2012) Ventromedial prefrontal-subcortical systems and the generation of affective meaning. Trends Cogn Sci 16: 147–156 10.1016/j.tics.2012.01.005 22310704 PMC3318966

[29] Northoff G, Bermpohl F (2004) Cortical midline structures and the self. Trends Cogn Sci 8: 102–107. 15301749 10.1016/j.tics.2004.01.004

[30] Krueger F, Barbey AK, Grafman J (2009) The medial prefrontal cortex mediates social event knowledge. Trends Cogn Sci 13: 103–109 10.1016/j.tics.2008.12.005 19223228

[31] Buckner RL, Carroll DC (2007) Self-projection and the brain. Trends Cogn Sci 11: 49–57. 17188554 10.1016/j.tics.2006.11.004

[32] Mahler SV, Smith KS, Berridge KC (2007) Endocannabinoid hedonic hotspot for sensory pleasure: anandamide in nucleus accumbens shell enhances ‘liking’ of a sweet reward. Neuropsychopharmacology 32: 2267–2278. 17406653 10.1038/sj.npp.1301376

[33] Carlson JM, Foti D, Mujica-Parodi LR, Harmon-Jones E, Hajcak G (2011) Ventral striatal and medial prefrontal BOLD activation is correlated with reward-related electrocortical activity: a combined ERP and fMRI study. Neuroimage 57: 1608–1616 10.1016/j.neuroimage.2011.05.037 21624476

[34] van den Bos W, Cohen MX, Kahnt T, Crone EA (2012) Striatum-medial prefrontal cortex connectivity predicts developmental changes in reinforcement learning. Cereb Cortex 22: 1247–1255 10.1093/cercor/bhr198 21817091 PMC6283353

[35] Sharot T, Riccardi AM, Raio CM, Phelps EA (2007) Neural mechanisms mediating optimism bias. Nature 450: 102–105. 17960136 10.1038/nature06280

[36] Etkin A, Egner T, Peraza DM, Kandel ER, Hirsch J (2006) Resolving emotional conflict: a role for the rostral anterior cingulate cortex in modulating activity in the amygdala. Neuron 51: 871–882. 16982430 10.1016/j.neuron.2006.07.029

[37] Etkin A, Egner T, Kalisch R (2011) Emotional processing in anterior cingulate and medial prefrontal cortex. Trends Cogn Sci 15: 85–93 10.1016/j.tics.2010.11.004 21167765 PMC3035157

[38] Petrides M, Pandya DN (2007) Efferent association pathways from the rostral prefrontal cortex in the macaque monkey. J Neurosci 27: 11573–11586. 17959800 10.1523/JNEUROSCI.2419-07.2007 PMC6673207

